# Institutional guidelines on maternal care and investigations following antepartum stillbirth - a national survey

**DOI:** 10.1186/s12884-021-03995-z

**Published:** 2021-07-24

**Authors:** Dana A. Muin, Sabrina Neururer, Veronika Rotter, Hermann Leitner, Stephanie Leutgeb, Peter W. Husslein, Herbert Kiss, Petra Kohlberger

**Affiliations:** 1 Department of Obstetrics and Gynecology, Division of Feto-Maternal Medicine, Medical University of Vienna, Waehringer Guertel 18-20, 1090 Vienna, Austria; 2 Department of Clinical Epidemiology, Tyrolean Federal Institute for Integrated Care, Tirol Kliniken GmbH, Anichstraße 35, 6020 Innsbruck, Austria; 3 Austrian Society of Obstetrics and Gynecology, Frankgasse 8, 1090 Vienna, Austria

**Keywords:** Stillbirth, Fetal death, Guideline, Post-mortem work-up

## Abstract

**Background:**

Antepartum stillbirth, i.e., intrauterine fetal death (IUFD) above 24 weeks of gestation, occurs with a prevalence of 2.4–3.1 per 1000 live births in Central Europe. In order to ensure highest standards of treatment and identify causative and associated (risk) factors for fetal death, evidence-based guidelines on clinical practice in such events are recommended. Owing to a lack of a national guideline on maternal care and investigations following stillbirth, we, hereby, sought to assess the use of institutional guidelines and clinical practice after IUFD in Austrian maternity units.

**Methods:**

A national survey with a paper-based 12-item questionnaire covering demographic variables, local facilities and practice, obstetrical care and routine post-mortem work-up following IUFD was performed among all Austrian secondary and tertiary referral hospitals with maternity units (*n* = 75) between January and July 2019. Statistical tests were conducted using Chi^2^ and Fisher’s Exact test, respectively. Univariate logistic regression analyses were performed to calculate odds ratio (OR) with a 95% confidence interval (CI).

**Results:**

46 (61.3%) obstetrical departments [37 (80.4%) secondary; 9 (19.6%) tertiary referral hospitals] participated in this survey, of which 17 (37.0%) have implemented an institutional guideline. The three most common investigations always conducted following stillbirth are placental histology (20.9%), fetal autopsy (13.1%) and maternal antibody screen (11.5%). Availability of an institutional guideline was not significantly associated with type of hospital, on-site pathology department, or institutional annual live and stillbirth rates. Post-mortem consultations only in cases of abnormal investigations following stillbirth were associated with lower odds for presence of such guideline *[OR 0.133 (95% CI 0.018–0.978); p* = *0.047]*. 26 (56.5%) departments consider a national guideline necessary.

**Conclusions:**

Less than half of the surveyed maternity units have implemented an institutional guideline on maternal care and investigations following antepartum stillbirth, independent of annual live and stillbirth rate or type of referral centre.

**Supplementary Information:**

The online version contains supplementary material available at 10.1186/s12884-021-03995-z.

## Background

Antepartum stillbirth, i.e., intrauterine fetal death (IUFD) above 24 weeks of gestation, occurs with a prevalence of 2.4–3.1 per 1000 live births in Central Europe [1, 2]. Due to underlying risk factors, recurrence risk may be as high as 20-fold in subsequent pregnancies [3]. A detailed and standardized work-up following stillbirth is therefore indicated in all cases to detect potential risk factors and define the cause of death [4, 5]. Medical guidelines and recommendations on clinical practice are usually published by national societies or networks [6]. Guidance on maternal care and investigations following antepartum stillbirth is available from societies as the *Royal College of Obstetricians and Gynaecologists* (RCOG), the *Perinatal Society of Australia and New Zealand* (PSANZ) and the *American College of Obstetrics and Gynecology* (ACOG) [7–10]. The main overarching components in these are the conduction of fetal autopsy, macroscopic and histological evaluation of the placenta and umbilical cord, as well as maternal examinations, such as the Kleihauer-Betke test, glucose tolerance test, thyroid function test, and screening for infections or thrombophilia. A thorough post-mortem work-up has shown to elucidate the aetiology in up to 90% of cases and should therefore be an indispensable element in care following antepartum stillbirth.

To date, none of the German-speaking fetal-maternal professional Societies (i.e., Austrian, German, Swiss) have published any official national protocol on care and investigations following stillbirth. Considering the option of local protocols at individual institutions, we, therefore, sought to explore how many Austrian secondary and tertiary referral hospitals with maternity units have implemented an institutional guideline on maternal care and investigation following stillbirth, and which factors night have perpetuated the implementation of such. Lastly, we aimed to assess local standards on maternal care and work-up following fetal death in all institutions to gain an insight into clinical practice across Austrian maternity units.

## Methods

### Data collection

We created a 12-item questionnaire covering demographic variables (3 items), local facilities and practice following IUFD (5 items), routine post-mortem work-up (1 item) and obstetrical care after IUFD (3 items; Additional file 1).

The demographic variables included hospital location as per Austrian federal region *(categorical variable; Vienna; Lower Austria; Upper Austria; Burgenland; Styria; Carinthia; Salzburg; Tyrol; Vorarlberg*), type of institution (*categorical variable; secondary referral hospital; tertiary referral hospital)* and annual live birth-rate (*categorical variable;* ≤ *500; 501 – 1000; 1001 – 2000;* ≥ *2001*).

One question regarding local facilities assessed whether there is a department of pathology on-site of the hospital (*dichotomous variable; yes; no*) and whether the institution has a guideline on maternal care and investigations following antepartum stillbirth (*dichotomous variable; yes; no*). In that context, we surveyed whether there was a felt need for a national guideline on clinical management after stillbirth in Austria (*dichotomous variable; yes; no).*

Practice following IUFD included the question, whether the department would admit the woman for induction of labour (IOL) or whether the woman would need to be transferred to another hospital for delivery (*dichotomous variable; yes; no*) and whether the institution would offer a post-mortem consultation following stillbirth delivery (*categorical variable; no; always; yes; if requested by the parents; if examinations were conducted; if results were suspicious/abnormal*).

One question regarding routine post-mortem work-up covered the types of investigations conducted following IUFD, proposing fetal autopsy, fetal magnetic resonance imaging (MRI), placental histology, fetal genetics (*e.g., chromosomal examination; microarray examination; whole exome sequencing*) and source of genetic material (*umbilical cord blood; amniotic fluid; fetal muscle biopsy; placental tissue*), and finally maternal examinations, such as Kleihauer testing, antibody screening, blood cultures for infections, virology, urine culture, vaginal swabs, HbA1c, glucose tolerance test, thyroid function test and thrombophilia screening (*categorical variables; always; under certain circumstances; never*).

Finally, we surveyed obstetrical care after IUFD: Firstly, we assessed timing of admission for labour induction (*categorical variable; straight after first diagnosis; on the following day; after two days; after 1 week; depending on woman’s preference; others*), and secondly, medication regime for IOL after fetal death (*categorical variable;* ≤ *27*^+*6*^* gestational weeks: mifepristone; misoprostol; others*; ≥ *28*^+*0*^* gestational weeks: mifepristone; misoprostol; dinoproston; balloon; others*).

Our final variable elaborated on psychological support for affected women after IUFD (*categorical variable; as an in-patient; as an outpatient; none; others*).

### Study design

The questionnaire was sent to all (*n* = 75) secondary and tertiary referral hospitals with a maternity unit in Austria and was accompanied by an invitation letter addressed to the head of the obstetric department and a franked response envelope. The heads of department or one of their designated consultants were asked to fill out and (anonymously) return the questionnaire. Survey time was between January and July 2019 and deadline was set after 3 months of receipt. A reminder was sent by email to the departmental director directly after 2 months.

Returned questionnaires were extracted for all variables and answers were transferred into an excel file sheet. Institutional epidemiological data were retrieved from the Department of Clinical Epidemiology Tirol Kliniken, Austria, and combined with our dataset. After data check for integrity and consistency, the database was frozen and made anonymous prior to final analysis.

### Statistical analyses

Descriptive categorical data are given as counts (n) and percentages (%). We compared categorical variables with Chi^2^ and Fisher’s Exact test, respectively. Associations between variables were assessed by Pearson’s correlations and Chi^2^ tests. Additionally, univariate binary logistic regressions were performed to assess the odds ratio (OR) and 95% Confidence Interval (CI) for variables with more than two categories. A forest plot with a logarithmic scale (natural logarithm) illustrates the OR and 95% CIs. All tests are two-sided, and the level of significance was set at < 0.05. Statistical tests were performed with SPSS Statistics Version 26.0 (IBM Corporation, Armonk, NY, USA) and figures designed by GraphPad Prism 9 for macOS (GraphPad Software, LLC) and STATA (16.0, StataCorp LLC, College Station, TX).

## Results

### Institutional demographics

In total, 46 (61.3%) maternity units from 37 (80.4%) secondary and 9 (19.6%) tertiary referral hospitals, respectively, participated in this survey. The respondents were from eight out of nine Austrian federal states [5 (10.9%) units from the capital Vienna; 16 (34.8%) from Lower Austria; 8 (17.4%) from Upper Austria; 6 (13.0%) from Tyrol; 4 (8.7%) from Styria; 3 (6.5%) from Vorarlberg; 2 (4.3%) from Salzburg and 2 (4.3%) from Carinthia]. Annual live birth rate was ≤ 500 in 5 (10.9%) participating hospitals, 501–1000 in 18 (39.1%) hospitals, 1001–2000 in 14 (30.4%) hospitals and ≥ 2001 in 9 (19.6%) hospitals.

#### Presence of institutional guidelines

**Fig. 1:**
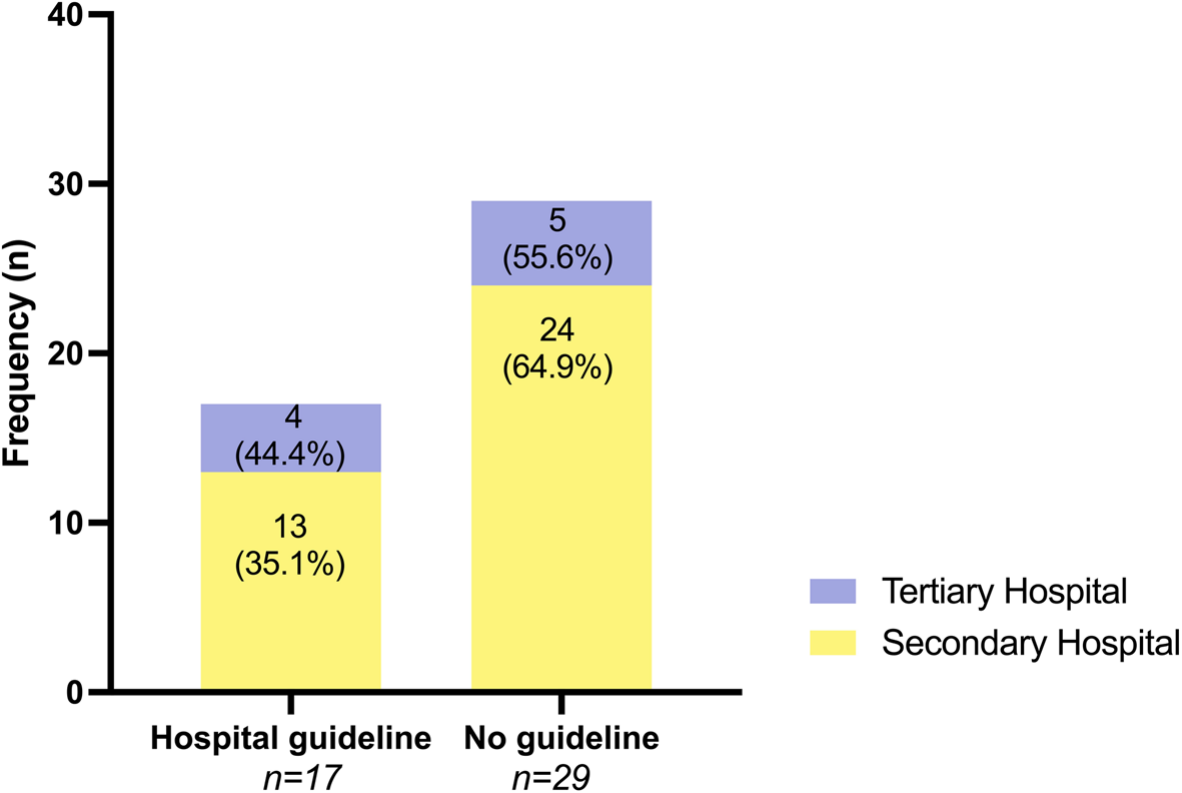
Presence of an institutional guideline on maternal care and investigations following stillbirth in Austrian secondary and tertiary referral hospitals (total *n* = 46)

17 (37.0%) maternity units [13 (35.1%) secondary vs. 4 (44.4%) tertiary referral hospitals] replied to have an institutional guideline on maternal care and investigations following stillbirth, whilst the majority of units (*n* = 29; 63.0%) responded to have none [24 (64.9%) secondary vs. 5 (55.6%) tertiary hospitals; Fig. 1].

Independent of the local implementation of an institutional guideline, 26 (56.5%) departments [22 (84.6%) secondary vs. 4 (44.4%) tertiary referral hospitals] consider a national guideline important and necessary, whilst 13 (28.3%) denied and 7 (15.2%) did not disclose their interest.

**Fig. 2:**
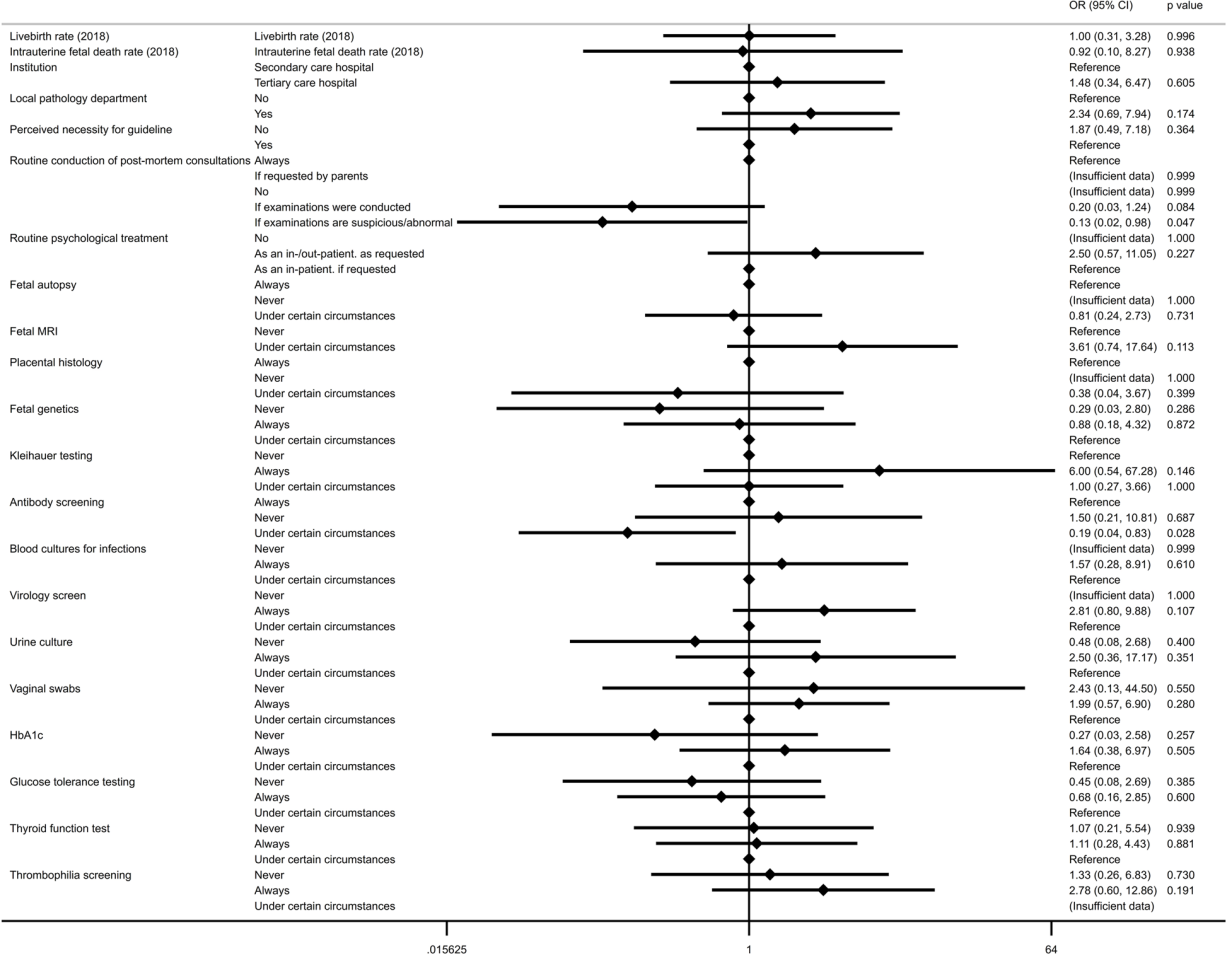
Forest plots illustrating the odds ratios (and 95% confidence intervals) from univariate binary logistic regression analyses identifying associations between availability of hospital guidelines and demographic characteristics and practice following intrauterine fetal death in maternity units in Austria (*n* = 46; level of significance *p* < 0.05, two-tailed)

The effect of demographic variables and local clinical standards on the implementation of an institutional guideline is illustrated in Fig. 2***.*** Whilst we found that, in our cohort, most maternity units with institutional guidelines were more likely to conduct post-mortem follow-up consultations with the parents after fetal death [*n* = 12; 70.6%; *χ*^*2*^*(7;40)* = *14.205; p* = 0.007], regression analysis indicated that follow-up consultations, scheduled only in case of suspicious or abnormal test results, were associated with lower odds for presence of an institutional guideline [*OR 0.133 (95% CI 0.018–0.978); p* = *0.047]*.

We also observed a trend in favour for a guideline with respect to the type of institution (i.e., university clinic), the presence of an affiliated pathology department, the recognition of the importance of psychological support, and the regular performance of post-mortem investigations, such as maternal screening for thrombophilia, diabetes or infection, though these effects did not reach statistical significance (Fig.  2).

####  Clinical practice on maternal care and investigations

#### Admission

Following diagnosis of fetal death, all maternity units (*n* = 46; 100%) would arrange IOL at their place. 19 (41.3%) units [16 (43.2%) secondary vs. 3 (33.3%) tertiary referral hospitals] would usually have the women decide when to be admitted to hospital for IOL. 8 (17.4%) units [6 (16.2%) secondary vs. 2 (22.2%) tertiary hospitals] usually admit the woman straight after diagnosis of fetal death, whereas 12 (26.1%) units [10 (27.0%) secondary vs. 2 (22.2%) tertiary hospitals] arrange re-admission on the subsequent day, 3 (6.5%) institutions [2 (5.4%) secondary vs. 1 (11.1%) tertiary hospitals] on the following day or the day after, and 4 (8.7%) units [3 (8.1%) secondary vs. 1 (11.1%) tertiary referral hospitals] after two days, as long as the woman remains clinically stable.

#### Methods for induction of labour

**Fig. 3:**
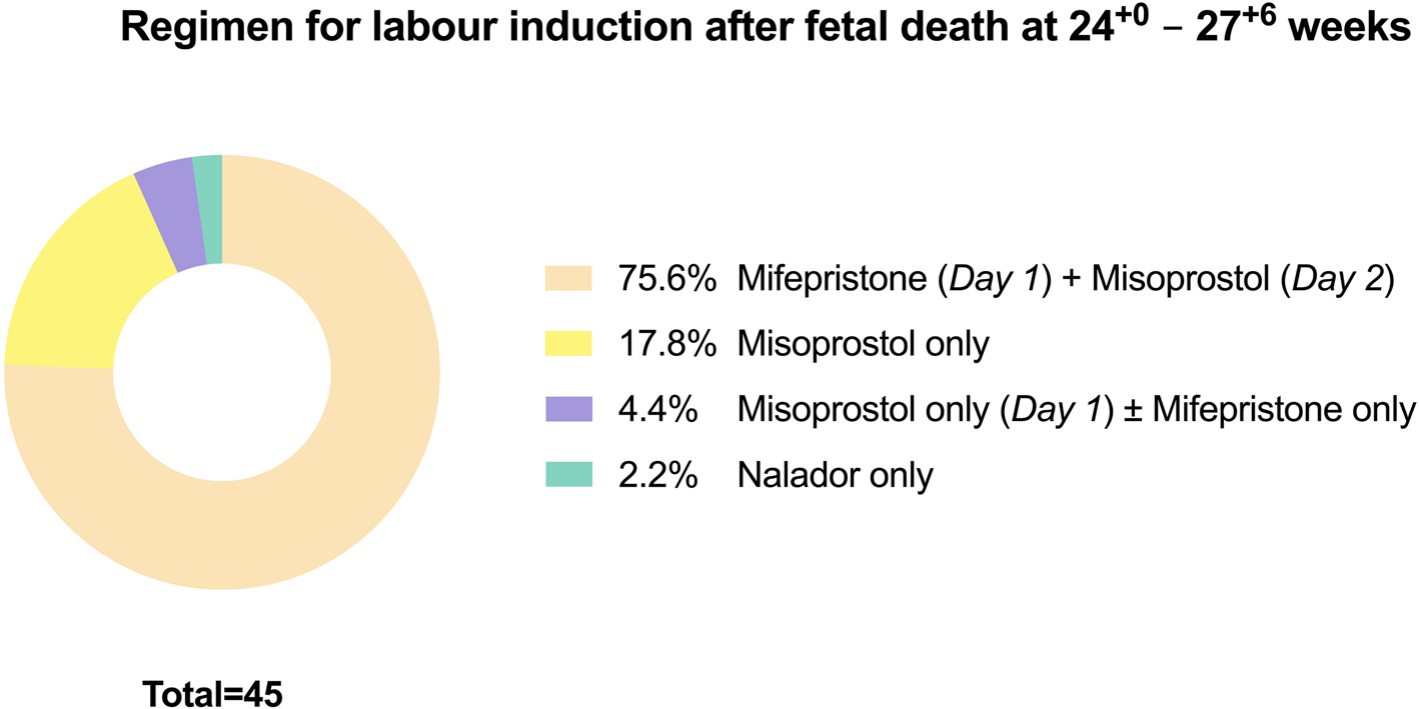
Regimen of induction of labour following antepartum stillbirth between 24^+0^ and 27^+6^ gestational weeks across Austrian maternity units (*multiple entries per institution possible*)

**Fig. 4:**
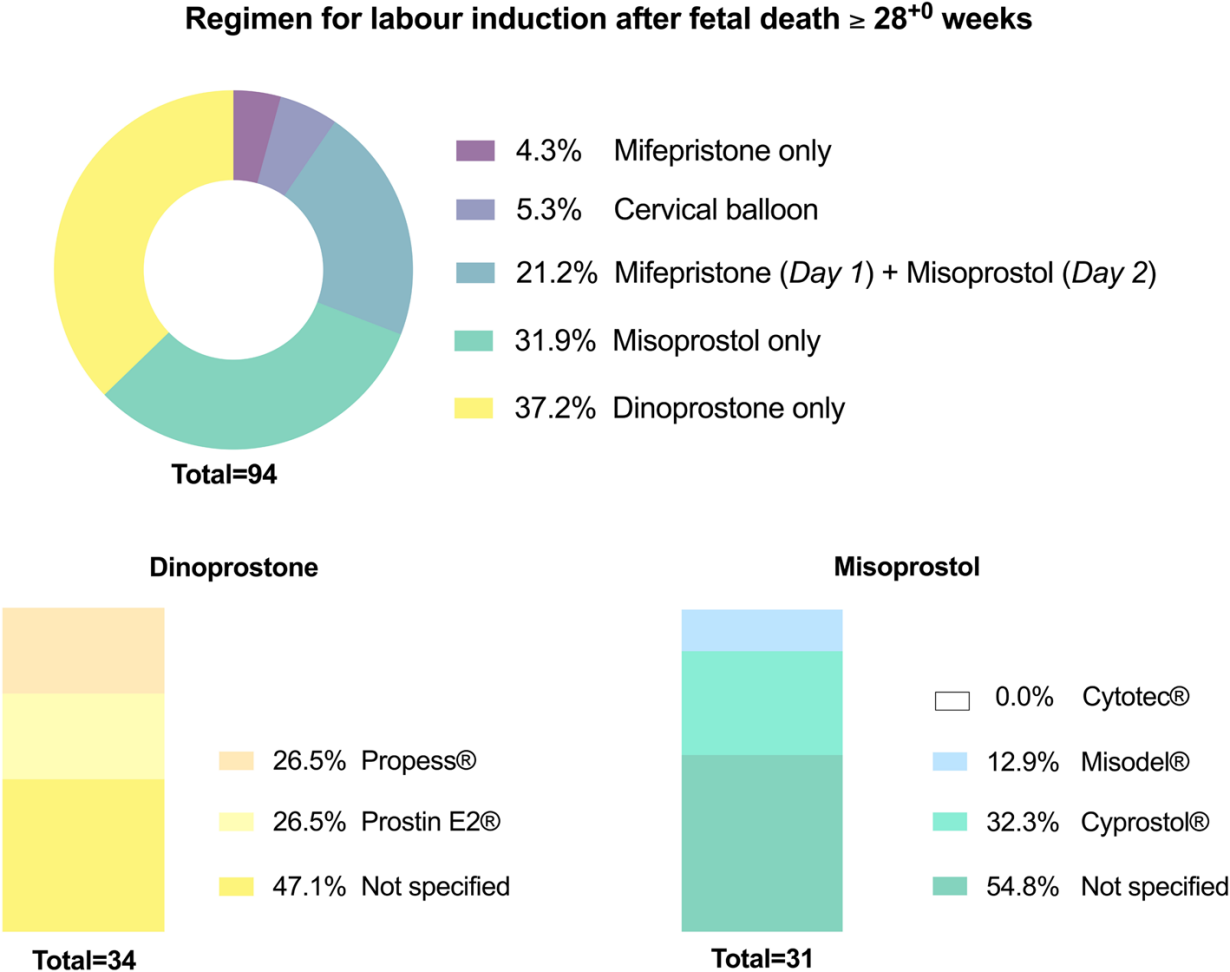
Regimen of induction of labour following antepartum stillbirth above 28^+0^ gestational weeks across Austrian maternity units (*multiple entries per institution possible*)

Across surveyed maternity units, most common regimen for IOL between 24^+0^ and 27^+6^ gestational weeks is mifepristone on admission day (i.e., Day 1), followed by misoprostol from second day onwards (Fig. 3). Above 28^+0^ gestational weeks, most institutions would induce their patients using the pharmacological substances misoprostol (31.9%) and dinoprostone (37.2%; Fig. 4).

#### Investigations following antepartum stillbirth

In 21 (45.7%) surveyed hospitals [13 (35.1%) secondary vs. 8 (88.9%) tertiary referral hospitals], a local pathology department is on site, whilst the majority of hospitals have none [24 (64.9%) secondary vs. 1 (11.1%) tertiary hospitals]. In Austria, presence of a local pathology department strongly correlates with the type of hospital (*p* = 0.003) and annual hospital live birth rate (*p* = 0.039). Also, hospitals with an integrated pathology department would perform fetal genetic tests following stillbirth more often, than units with no integrated pathology department (*p* = 0.017).

Maternity units, which usually admit the woman immediately after diagnosis of fetal death, are more likely to perform maternal tests to rule out clotting or bleeding disorders, such as thrombophilia (*p* = 0.03) and occult feto-maternal haemorrhage by Kleihauer-Betke test (*p* = 0.049).

Table 1 summarizes all types of conducted maternal, placental and fetal post-mortem examinations following fetal death, independent of availability of an institutional guideline, and compares their frequency between secondary and tertiary care hospitals. Whilst 44.4% of tertiary referral hospitals consider requesting a fetal MRI after fetal death, 89.2% of secondary referral hospitals would never request such (*p* = 0.036). Likewise, significantly more tertiary referral hospitals would initiate chromosomal and microarray analyses following fetal death, than secondary referral hospitals (44.4% vs. 19.4%; *p* = 0.016). No other significant differences in regular post-mortem examinations were observed between secondary and tertiary referral hospitals.

**Table 1:** Summary on spectrum and frequency of performed fetal, placental and maternal post-mortem examinations following fetal death in secondary and tertiary care hospitals (*n* = 46) across Austria (frequency n; percentage %) and comparison of frequency between types of hospital (two-sided Fisher’s Exact test; level of significance *p* < 0.05)

Examinations			Total		Secondary care hospital		Tertiary care hospital		*P*-Value
			n	%	n	%	n	%	
Fetal	**Autopsy**	*Always*	25	54.3	19	51.4	6	66.7	0.663
		*Under certain circumstances*	20	43.5	17	45.9	3	33.3	
		*Never*	1	2.2	1	2.7	0	0.0	
	**MRI**	*Always*	0	0.0	0	0.0	0	0.0	0.036
		*Never*	38	82.6	33	89.2	5	55.6	
		*Under certain circumstances*	8	17.4	4	10.8	4	44.4	
	**Genetics**	*Always*	8	17.4	7	18.9	1	11.1	0.314
		*Under certain circumstances*	32	69.6	24	64.9	8	88.9	
		*Never*	6	13.0	6	16.2	0	0.0	
	**Type of genetic test**	*Karyotype*	21	52.5	19	61.3	2	22.2	0.016
		*Karyotype* + *Microarray*	10	25.0	6	19.4	4	44.4	
		*Microarray only*	1	2.5	1	3.2	0	0.0	
		*Not disclosed*	8	20.0	5	16.1	3	33.3	
Placenta	**Histology**	*Always*	40	87.0	33	89.2	7	77.8	0.397
		*Under certain circumstances*	5	10.9	3	8.1	2	22.2	
		*Never*	1	2.2	1	2.7	0	0.0	
Maternal	**Kleihauer**	*Always*	4	8.7	4	10.8	0	0.0	0.871
		*Under certain circumstances*	18	39.1	14	37.8	4	44.4	
		*Never*	24	52.2	19	51.4	5	55.6	
	**Antibody screening**	*Always*	22	47.8	17	45.9	5	55.6	0.871
		*Under certain circumstances*	19	41.3	16	43.2	3	33.3	
		*Never*	5	10.9	4	10.8	1	11.1	
	**Blood cultures**	*Always*	6	13.0	6	16.2	0	0.0	0.540
		*Under certain circumstances*	36	78.3	28	75.7	8	88.9	
		*Never*	4	8.7	3	8.1	1	11.1	
	**Virology screen**	*Always*	17	37.0	13	35.1	4	44.4	0.769
		*Under certain circumstances*	28	60.9	23	62.2	5	55.6	
		*Never*	1	2.2	1	2.7	0	0.0	
	**Urine culture**	*Always*	5	10.9	5	13.5	0	0.0	0.604
		*Under certain circumstances*	32	69.6	24	64.9	8	88.9	
		*Never*	9	19.6	8	21.6	1	11.1	
	**Vaginal swabs**	*Always*	20	43.5	16	43.2	4	44.4	1.000
		*Under certain circumstances*	24	52.2	19	51.4	5	55.6	
		*Never*	2	4.3	2	5.4	0	0.0	
	**HbA1c**	*Always*	10	21.7	9	24.3	1	11.1	0.765
		*Under certain circumstances*	29	63.0	22	59.5	7	77.8	
		*Never*	7	15.2	6	16.2	1	11.1	
	**oGTT**	*Always*	12	26.1	11	29.7	1	11.1	0.144
		*Under certain circumstances*	26	56.5	18	48.6	8	88.9	
		*Never*	8	17.4	8	21.6	0	0.0	
	**Thyroid function test**	*Always*	13	28.3	11	29.7	2	22.2	0.785
		*Under certain circumstances*	25	54.3	19	51.4	6	66.7	
		*Never*	8	17.4	7	18.9	1	11.1	
	**Thrombophilia screening**	*Always*	9	19.6	9	24.3	0	0.0	0.163
		*Under certain circumstances*	29	63.0	21	56.8	8	88.9	
		*Never*	8	17.4	7	18.9	1	11.1	

*Abbreviation*s: *HbA1c* Glycosylated Hemoglobin Type A1C, *MRI* Magnetic resonance imaging, *oGTT *Oral glucose tolerance test

**Fig. 5:**
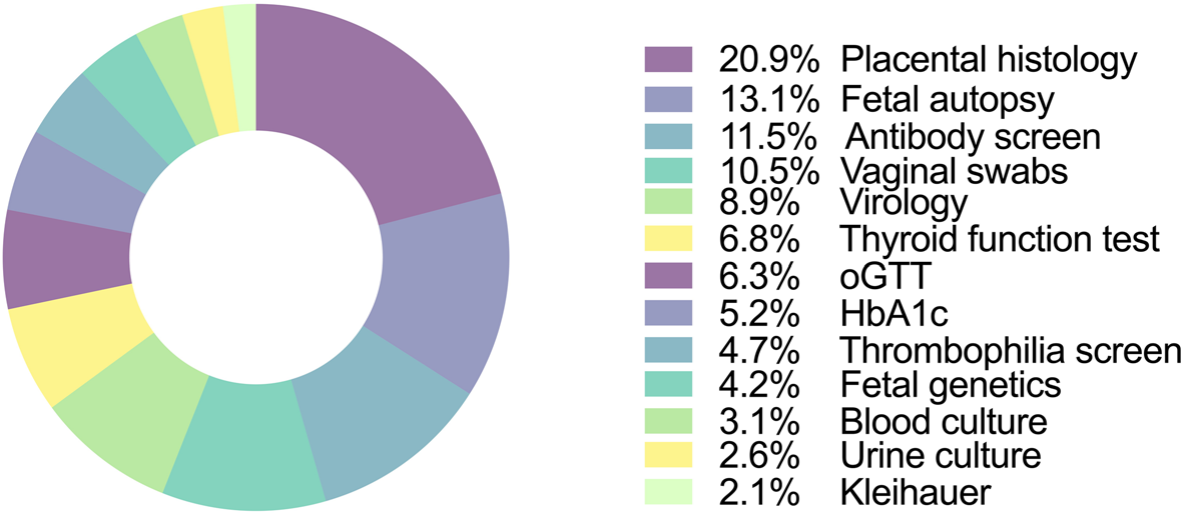
Overview on post-mortem examinations *always* performed after fetal death in maternity units, which took part in this survey

In summary, across Austrian maternity units, the three most commonly conducted investigations following antepartum stillbirth are placental histology (20.9%), fetal autopsy (13.1%) and maternal antibody screen (11.5%; Fig. 5). Fetal autopsy was strongly linked to fetal genetic analyses (*p* = 0.015) and placental histology (*p* = 0.031), yet was independent of a local pathology department (*p* = 0.341) and type of institution (*p* = 0.451).

##### Psychological support

All, but one surveyed maternity units would routinely offer professional psychological support to affected parents whilst hospitalized, and 9 (19.6%) departments [6 (16.2%) secondary vs. 3 (33.3%) tertiary referral hospitals] would also arrange psychological follow-up consultations as an outpatient.

##### Post-mortem follow-up consultations

16 (34.8%) units [12 (32.4%) secondary vs. 4 (44.4%) tertiary hospitals] replied to always schedule a regular post-mortem follow-up consultation with the parents following antepartum stillbirth, whilst 7 (15.2%) units [5 (13.5%) secondary vs. 2 (22.2%) tertiary hospitals] would never arrange such. In 8 (17.4%) units [6 (16.2%) secondary vs. 2 (22.2%) tertiary referral hospitals] post-mortem consultations would only be scheduled, if post-mortem investigations had been conducted. In 7 (15.2%) units, follow-up consultations would only be arranged in case of suspicious or abnormal test results, and in 2 (4.3%) institutions, only if requested by the parents. 6 (13.0%) units did not declare their practice after stillbirth.

## Discussion

In the absence of national clinical practice guidelines on maternal care and investigations following antepartum stillbirth in Central Europe, this is the first survey to investigate the implementation of institutional protocols in Austrian hospitals. With a response rate of 61 percent covering for 73% of all registered stillbirths and 64% of all registered live births in this country, exceeding the average response rate in postal surveys in healthcare professionals, [11] our survey provides a representative overview on practice and position.

As only a small proportion of hospitals had implemented local guidelines on maternal care and investigations following stillbirth, the robustness of our analyses to recognize obvious influential factors supporting such protocol was small. Whilst we observed a trend in favour for tertiary referral hospitals, affiliated pathology departments, and hospitals, which routinely offer psychological support during hospitalisation, we clearly found that units that only perform follow-up consultations in case of abnormal post-mortem investigations only, were less likely to have implemented an institutional guideline. After all, we have identified a common need for a national protocol, which may accelerate the implementation of such guideline by respective societies in the near future.

Interestingly, independent of the presence of a local guideline, we found that placental histology, fetal autopsy and maternal antibody screening were the three most common investigations requested after fetal death. In the United Kingdom (UK), fetal post-mortem examinations are carried out in about 44% of stillbirths, [12] and in the United States, the uptake of autopsy has been reported as low as 35% in tertiary care centres and 13% in community hospitals [13]. After all, autopsy and placental histology are considered the gold standards following fetal loss, as they give insight into the aetiology in 22–76% of the cases [14, 15].

Our study is not devoid of limitations inherent to the failure to control for recall bias of responders and thus data accuracy from returned questionnaires, potentially limiting their validity. As over half of the responding institutions showed a lack of a local guideline, yet their support for a national protocol, we cannot rule out a certain degree of selection bias. Also, hospitals with small numbers of stillbirths might not have participated in this survey. Furthermore, we have not included the post-mortem assessment of fetal carboxy-haemoglobin as a marker for chronic hypoxia related to maternal nicotine abuse, despite the prevalence of daily smoking habits in 27.5% of the Austrian female population aged 30 and 45 years [16]. Finally, our data might not be generalizable and translatable to other countries due to divergent facilities and different medico-legal practice following perinatal death. These limitations confirm the need to gain insight into care and practice following stillbirth in other European countries, too, to further strengthen our findings.

Despite these limitations, the high response rate in our survey results in a representative overview on clinical practice across the country [11]. Of note, all obstetric departments from medical university clinics participated in our survey, which demographically yield the highest numbers of annual stillbirth deliveries. Furthermore, the majority of returned questionnaires were fully completed, thus limiting the number of missing data. The absence of a formal local guideline does not rule out any other form of standard practice within an institution, as habits may be verbally shared and communicated within a team. The strength of the questionnaire was to capture these practices and provide an overview on clinical care in a cost-effective, reliable and versatile manner. The combination of institutional data from questionnaires with epidemiological data from the Austrian Birth Registry allowed us to interpret the results with greater detail by adjusting for the numbers of live and stillbirths per institution.

*Clinical Practice Guidelines* are “statements that include recommendations intended to optimize patient care” [17]. Whilst their aim is to enhance the medical practice of doctors and guide them along with evidence-based knowledge towards better patients’ care,[18] studies have likewise revealed poor adherence in cases where guidelines are not based upon good evidence,[19] lead to over-treatment rather than “effective” treatment of a patient,[20] or when conflicts of interests of authors create a bias in clinical recommendations [21, 22]. Defining the cause of death helps bereaved parents in their grieving process,[23, 24] estimates the recurrence risk in future pregnancies, serves mortality statistics and future public health interventions to reduce the number of perinatal loss [25]. Elucidating the cause and underlying risk factors through thorough and concise post-mortem investigations may therefore prevent future losses.

Evidence-based clinical practice guidelines on maternal and fetal post-mortem examinations therefore ought to be implemented in every hospital in order to optimize, standardize and harmonize their practice according to national guidelines such a supported by the RCOG, PSANZ or ACOG with the aim of finding the cause of fetal death and improve maternal care in subsequent pregnancies. Ideally, sensitivity of post-mortem examinations should be high with a low inter-rater variability in result interpretation in order to determine the underlying aetiology with high accuracy [8, 26]. Placental histopathology, fetal autopsy and fetal genetic testing are considered the gold standards following stillbirth [27]. Known barriers to post-mortem examinations, however, are considered parents’ dislike of invasiveness, poor communication and lack of understanding of the purpose [28]. Contrarily, parents’ desire for more information acts as a facilitator and it therefore lies within the responsibility of the caring obstetrician to propose the optimal post-mortem workup after stillbirth in a respectful way to bereaved parents. Obstetricians most commonly consent bereaved parents for perinatal autopsy, yet about 12.4% claim to lack training in counselling, after all [29].

Although the annual stillbirth rate has held stable at around 3.1 cases per 1000 live births beyond 24 gestational weeks for the last 12 years, in Austria,[1] a reduction of 10–15% by 2025 is desirable. In the UK, the annual stillbirth rate of 4.7 per 1000 live births ranks among the highest in Europe, which led the UK Department of Health and Social Care support the *National Health Service* to reduce the stillbirth rates by 50% by 2025. In 2015, the national prevention program “Saving Babies’ Lives Care Bundle” has been introduced, comprising four measures: (a) reduction of smoking in pregnancy, (b) assessment of risk and surveillance in growth restricted fetuses, (c) raising awareness of reduced fetal movements and (d) effective monitoring during labour [30]. Subsequently, UK hospitals had to implement local maternity guidelines in order to follow these steps towards better maternal and fetal care and prevention of stillbirth. A recent study evaluated the local practice guidelines from 75 participating UK hospitals and found that whilst only the minority of 5.6% of evaluated guidelines were recommended for clinical use and 75% needed some modifications, 16.7% were not recommended at all [31]. Assessment of staff opinions on the use of their clinical guidelines revealed that over half considered the guidelines to offer higher quality care to women, yet 30% of staff claimed not to be able to follow their guidelines due to time issues, while 24% were not able to implement their recommendations at all. These limitations clearly indicate that the quality, content and perceived utility of guidelines need to be addressed internationally.

## Conclusion

This national survey reveals that less than half of the surveyed maternity units have implemented an institutional guideline on maternal care and investigations following antepartum stillbirth, irrespective of annual live and stillbirth rate or type of referral centre. Independent of the implementation of a local guideline, there is a perceived need for a national guideline, which may ensure highest level of care and optimal post-mortem work-up following antepartum stillbirth across the country.

## Supplementary Information

**Additional file 1**. 12-item questionnaire sent out to 75 maternity units in Austria between January and July 2019 (Translated from German into English).

## Data Availability

The datasets used and analysed during the current study are available from the corresponding author on reasonable request.

## Abbreviations

IUFD: Intrauterine fetal death
MRI: Magnetic resonance imaging
ACOG: American College of Obstetrics and Gynecology
CI: Confidence Interval
IOL: Induction of labour
OR: Odds ratio
PSANZ: Perinatal Society of Australia and New Zealand
RCOG: Royal College of Obstetricians and Gynaecologists
UK: United Kingdom
