# Supplementary material for: Institutional guidelines on maternal care and investigations following antepartum stillbirth - a national survey
Source: BMC Pregnancy Childbirth. 2021 Jul 24;21:528. doi: 10.1186/s12884-021-03995-z (PMC8305903; doi:10.1186/s12884-021-03995-z)
Supplement: Supplementary file 1 — Additional file 1. 12-item questionnaire sent out to 75 maternity units in Austria between January and July 2019 (Translated from German into English). [file 12884_2021_3995_MOESM1_ESM.docx]

EPIDEMIOLOGY- INSTITUTION

*Please choose as appropriate:*

**Federal State**

- Vienna
- Lower Austria
- Upper Austria
- Burgenland
- Styria
- Carinthia
- Salzburg
- Tyrol
- Vorarlberg

**Institution**

- Secondary referral hospital
- Tertiary referral hospital

**Annual live birthrate**

- ≤ 500
- 501 - 1000
- 1001 - 2000
- ≥ 2001

LOCAL FACILITIES AND PRACTICE

**Would you perform the delivery of a stillborn fetus at your hospital after diagnosing the intrauterine fetal death or transfer the woman to another hospital for delivery?**

- Deliver at local hospital
- Transfer woman to another hospital
- Others: __________________________

**Is there a department of pathology at your hospital?**

- Yes
- No

**Do you offer a post-mortem consultation following delivery?**

- No
- Always
- Yes, only if (postmortem) examinations have been conducted
- Yes, only if postmortem examinations were suspicious/abnormal
- Others: __________________________

**Do you consider a lack of a national guideline regarding post-mortem examinations following IUFD?**

- Yes
- No

**Is there a local guideline regarding post-mortem examinations following IUFD at our institution?**

- Yes
- No

POST-MORTEM WORK-UP

**Which post-mortem examinations would you conduct following IUFD?**

1. Fetal autopsy

☐ Always ☐ Under certain circumstances ☐ Never

1. Fetal MRI

☐ Always ☐ Under certain circumstances ☐ Never

1. Placental histology

☐ Always ☐ Under certain circumstances ☐ Never

1. Fetal genetics

☐ Always ☐ Under certain circumstances ☐ Never

- 1. Methods

☐ Chromosomal examination

☐ Microarray examination

☐ Whole Exome Sequencing

- 1. Source of genetic material

☐ Umbilical cord blood

☐ Amniotic fluid

☐ Fetal muscle biopsy

☐ Placental tissue

1. Maternal examinations:

Kleihauer testing

☐ Always ☐ Under certain circumstances ☐ Never

Antibody Screening

☐ Always ☐ Under certain circumstances ☐ Never

Blood cultures for infections

☐ Always ☐ Under certain circumstances ☐ Never

Virology

☐ Always ☐ Under certain circumstances ☐ Never

Urine culture

☐ Always ☐ Under certain circumstances ☐ Never

Vaginal swabs

☐ Always ☐ Under certain circumstances ☐ Never

HbA1c

☐ Always ☐ Under certain circumstances ☐ Never

oGTT

☐ Always ☐ Under certain circumstances ☐ Never

Thyroid function test

☐ Always ☐ Under certain circumstances ☐ Never

Thrombophilia screening

☐ Always ☐ Under certain circumstances ☐ Never

OBSTETRICAL CARE

**Do you keep the woman as an in-patient straight after first diagnosis of IUFD or allow readmission?**

- keep the woman as an in-patient straight after first diagnosis
- As long as woman is clinically stable, allow readmission:
  - on following day
  - after two days
  - after 1 week
  - Others: __________________________
- Depending on woman’s preference
- Others: __________________________

**Medication used to induce labour in IUFD:**

1. Between 24^+0^/40 und 27^+6^/40

- Mifepristone only line
- Misoprostol only line
- Day 1 Mifepristone, Day 2 (+3) Misoprostol
- Others: __________________________

1. After SSW 28^+0^

- Mifepristone only line
- Day 1 Mifepristone, Day 2 (+3) Misoprostol
- Misoprostol
  - Cyprostol®
  - Cytotec®
  - Misodel®
- Dinoproston
  - Propess®
  - Prostin E2®
- Balloon
- Others: __________________________

**Do you routinely offer psychological treatment to women after diagnosis of IUFD?**

- Yes, as an in-patient, if requested by the woman
- Yes, in the outpatient setting, if requested by the woman
- No
- Others: __________________________
